# The PATHFAST TB LAM Ag assay: a validated protocol for quantitative tuberculosis treatment monitoring

**DOI:** 10.3389/frabi.2026.1844052

**Published:** 2026-07-28

**Authors:** Yassir Adam Shuaib, Stéphane Coisne

**Affiliations:** 1 PHC Europe, Breda, Netherlands; 2 College of Veterinary Medicine, Sudan University of Science and Technology, Khartoum North, Sudan

**Keywords:** lipoarabinomannan (LAM), PATHFAST, chemiluminescent enzyme immunoassay (CLEIA), tuberculosis, treatment monitoring, protocol

## Abstract

Tuberculosis (TB) is a global health challenge and is curable, but tools for rapid treatment monitoring are limited. Current methods, like smear microscopy, lack sensitivity, while culture-based approaches require weeks for results and specialized biosafety facilities. This paper presents a comprehensive protocol for the PATHFAST TB LAM Ag assay, a rapid chemiluminescent enzyme immunoassay that quantifies lipoarabinomannan (LAM) mainly in sputum and likely other biological samples within one hour. This protocol details sample collection, heat inactivation for biosafety, and the automated measurement process. Heat inactivation at 100 °C for 20 minutes renders samples non-infectious while preserving LAM for detection. The assay demonstrates excellent analytical performance with a detection limit of 6.67 pg/mL and a linear measurement range of 10.0–50,000 pg/mL. Published clinical evaluation demonstrated 88.8% (95% CI: 80.0–94.0%) sensitivity and 100% (95% CI: 83.9–100%) specificity compared to culture, with strong correlation to bacterial load. The PATHFAST TB LAM Ag assay successfully tracks bacterial decline during treatment, with LAM concentration reductions mirroring culture results but available significantly faster. Unlike culture methods that suffer from contamination (4.2-9.6% invalid results), the PATHFAST TB LAM Ag assay consistently provides valid results. This protocol enables implementation of a rapid, quantitative TB treatment monitoring tool that enhances biosafety, reduces turnaround time, and maintains performance comparable to conventional methods, addressing critical needs in both clinical care and drug development settings.

## 1 Introduction

Tuberculosis (TB) is caused by members of the *Mycobacterium tuberculosis* Complex (MTBC). It remains a formidable global health crisis and demands urgent advancements in diagnostic and monitoring technologies (WHO, 2025). The WHO Global Tuberculosis Report 2025 estimates 10.7 million new TB cases and 1.23 million deaths in 2024 (WHO, 2025). The burden of TB is disproportionately borne by low- and middle-income countries (LMICs). These countries account for over 80% of all TB cases highlighting the critical need for diagnostic and treatment monitoring tools that are not only accurate and rapid but also affordable, accessible, and resilient enough to be deployed in resource-constrained settings. The WHO’s End TB Strategy has set ambitious goals to reduce TB incidence by 90% and deaths by 95% by 2035 (WHO, 2015). This goal hinges on the development and implementation of innovative tools to improve every step of the TB care cascade from diagnosis to treatment completion. Effective treatment monitoring, in particular, is a cornerstone of this strategy, as it enables clinicians to optimize patient outcomes, prevent the emergence and spread of drug resistance, and accelerate the evaluation of new therapeutic regimens.

However, the current armamentarium of TB treatment monitoring are facing significant limitations that impede effective patient management and hinder progress in global TB control. Sputum smear microscopy, the most widely used method in many parts of the world, is inexpensive but suffers from poor sensitivity (50–60%) especially in patients with low bacterial loads and HIV co-infection (Steingart et al., 2006; Getahun et al., 2007; Perkins and Cunningham, 2007; Lawn et al., 2012; Shuaib et al., 2018). Mycobacterial culture, while considered the gold standard for both diagnosis and monitoring, is slow, with results taking anywhere from two to eight weeks (Wallis et al., 2010, 2013; Gillespie et al., 2014; Imperial et al., 2018). This prolonged turnaround time creates a critical delay in clinical decision-making which can have severe consequences for patients and public health. Furthermore, culture-based methods require sophisticated Biosafety Level 3 (BSL-3) laboratory infrastructure and are prone to contamination with rates as high as 10% leading to invalid results (Cruciani et al., 2004). These requirements make culture-based monitoring impractical for routine use in many high-burden settings. Molecular methods, such as the Xpert MTB/RIF assay, have revolutionized TB diagnosis but have limited utility for treatment monitoring because they target DNA sequences that can persist in non-viable bacterial cells, making it difficult to reliably assess microbiological treatment response, although the kinetics of DNA clearance vary and this limitation is context-dependent (Gillespie et al., 2014; Imperial et al., 2018). Clinical and radiological assessments, while important, lack the specificity needed to track microbiological response precisely, as symptoms and imaging changes often lag behind bacterial clearance.

Among emerging quantitative monitoring tools, the Molecular Bacterial Load Assay (TB-MBLA) represents the closest molecular comparator for quantitative bacterial load monitoring during TB treatment (Sabiiti et al., 2020; Musisi et al., 2022; Gupta-Wright et al., 2023). TB-MBLA targets mycobacterial 16S rRNA, providing a viability-linked signal with a wider dynamic range (Sabiiti et al., 2020; Musisi et al., 2022; Gupta-Wright et al., 2023). However, TB-MBLA currently remains in late-stage development, requires an approximately 4-hour workflow at district laboratory level, and is not yet commercially available for clinical implementation (Branigan, 2025). According to the TAG 2025 Pipeline Report, the PATHFAST TB LAM Ag assay is currently the only commercially available quantitative bacterial load monitoring tool (Branigan, 2025).

These challenges highlight the urgent need for a new generation of biomarkers and diagnostic tools that can provide rapid, accurate, and quantitative assessment of bacterial load. Lipoarabinomannan (LAM) is a major glycolipid component of the MTBC cell wall. It has lately emerged as a highly promising biomarker in TB diagnostics (Lawn and Gupta-Wright, 2016). LAM is specific to mycobacteria, abundant in infected individuals, and is released from bacterial cells during active disease and in response to effective treatment. Therefore, it is a dynamic indicator of bacterial burden. LAM can also be detected in various clinical specimens, such as sputum and urine, broadening its potential clinical applications (Nakiyingi et al., 2015; Lawn and Gupta-Wright, 2016; Lawn et al., 2017; Sigal et al., 2018; Broger et al., 2019; Bjerrum et al., 2019; Kerkhoff et al., 2020). While urine-based LAM tests have proven useful for diagnosing TB in specific populations, particularly people living with HIV, their qualitative or semi-quantitative nature limits their utility for monitoring treatment response. Sputum LAM, on the other hand, has shown a strong correlation with bacterial load and treatment response in patients with pulmonary TB (Mukundan et al., 2012; Kawasaki et al., 2019; Orina et al., 2025; Fouchet et al., 2025). The TB LAM ELISA “Otsuka” demonstrated that sputum LAM concentrations decline in proportion to the reduction in bacterial load during therapy, but its practical application is limited by a lengthy procedure (>5 hours) and a narrow dynamic range.

To overcome these hurdles, the PATHFAST TB LAM Ag assay, a novel chemiluminescent enzyme immunoassay (CLEIA), has been developed. This assay is designed to provide rapid, quantitative measurement of LAM in sputum samples, offering a significant leap forward in TB treatment monitoring. It utilizes the automated the PATHFAST platform which is a bench-top analyzer to deliver results within one hour including a crucial heat inactivation step that renders the sample non-infectious, thereby reducing biosafety requirements and allowing for safer handling in a wider range of laboratory settings (Akinaga et al., 2024). The PATHFAST TB LAM Ag assay offers a wide dynamic range (10.0–50,000 pg/mL), enabling precise quantification of LAM across a broad spectrum of bacterial loads. This protocol provides a comprehensive, standardized methodology for the PATHFAST TB LAM Ag assay, from sample collection and processing to result interpretation (Akinaga et al., 2024; Pichlo et al., 2025). The development of this protocol is guided by the WHO’s Target Product Profiles (TPPs) for tests to monitor TB treatment response, which call for rapid, quantitative, and operationally simple tools (WHO, 2023). By providing a standardized and validated protocol, this paper aims to facilitate the global adoption of this innovative technology, empowering clinicians and researchers with a powerful new tool to combat the global TB epidemic.

The objective of this manuscript is to provide a comprehensive, standardized, step-by-step protocol for the PATHFAST TB LAM Ag assay, integrating validated pre-analytical requirements, CLEIA measurement procedure, performance characteristics, and interpretation guidance. The protocol synthesizes procedural knowledge from the primary validation study and incorporates subsequently published pre-analytical findings to provide the most current and complete implementation guide for this assay (Akinaga et al., 2024; Pichlo, 2025; Pichlo et al., 2025).

## 2 Materials

### 2.1 Reagents and materials

A number of specialized reagents and materials are essential for the accurate and reliable execution of the PATHFAST TB LAM Ag assay. While key components are provided within the PATHFAST kit, certain common laboratory reagents and consumables must be procured externally (**Table 1**).

**Table 1:** Reagents and consumables for the PATHFAST TB LAM Ag assay.

Item	Manufacturer/supplier	Catalogue number	Purpose/notes
Reagent cartridges	PHC Corporation	PF1231-K	Contains all necessary reagents for 60 tests including magnetic beads, detection antibodies, and substrate
Calibrator set	PHC Corporation	Included in the kit	Two levels (CAL-1 and CAL-2) for generating a standard curve and ensuring accurate quantification
MC ENTRY CARD	PHC Corporation	Included in the kit	Used to input master calibration information into the PATHFAST analyzer
Control set	PHC Corporation	PF1231C	Two levels (2ml, 3 bottles) for daily quality control and monitoring assay performance
Sample Diluent 1	PHC Corporation	PF01D	Used for the samples with the results greater than 50,000 pg/mL
Sodium hydroxide (NaOH), 1.0 N	e.g., Sigma-Aldrich	S2770	CAS 1310-73-2. Used for sputum liquefaction
Sodium phosphate monobasic (NaH_2_PO_4_), 5 M	e.g., Sigma-Aldrich	74092	CAS 7558-80-7. Used for neutralizing sputum samples after NaOH treatment

### 2.2 Equipment

Beyond the assay-specific reagents, the PATHFAST TB LAM Ag protocol necessitates a suite of dedicated equipment to facilitate accurate sample processing and automated analysis. This includes the central PATHFAST Immunoanalyzer alongside standard laboratory apparatus required for sample preparation and biosafety (**Table 2**).

**Table 2:** Essential equipment for the PATHFAST TB LAM Ag assay.

Item	Manufacturer/supplier	Catalogue number	Purpose/notes
PATHFAST analyzer	PHC Corporation	300929	Automated bench-top analyzer for chemiluminescent enzyme immunoassay (CLEIA)
PATHFAST pipette tips	PHC Corporation	300936	Dedicated disposable tips for the PATHFAST Immunoanalyzer (5 x 42 units per pack)
PATHFAST waste box	PHC Corporation	300950	Container for safe disposal of used PATHFAST pipette tips from the analyzer (10 units per pack)
High-density liquids pipette and tips	e.g., Microman M1000E or Gilson M250E	N/A	Essential for accurate and precise transfer of viscous sputum samples
Microtube heater plate	Various laboratory suppliers	N/A	Capable of maintaining 100 °C for heat inactivation of samples
Microtube centrifuge	Various laboratory suppliers	N/A	Capable of 3,000 × g for separating sample components after liquefaction
Vortex mixer	Various laboratory suppliers	N/A	For thorough mixing of reagents and samples
Pipettes	Various laboratory suppliers	N/A	Adjustable volume pipettes (100 µL and 200 µL) for precise liquid handling
Pipette tips	Various laboratory suppliers	N/A	Disposable tips for 100 µL and 200 µL pipettes
Sterile, conical 1.5 mL or 2.0 mL screw-cap microtubes	Various laboratory suppliers	N/A	For safe handling, processing, and heat inactivation of samples
Timer	Various laboratory suppliers	N/A	For accurate timing of incubation and reaction steps
Discard container	Various laboratory suppliers	N/A	For safe disposal of biohazardous waste
Biosafety cabinet	Various laboratory suppliers	N/A	Class II or higher recommended for safe handling of potentially infectious samples

## 3 Methods

### 3.1 Validation

The comprehensive validation of this method was meticulously conducted to assess both its analytical and clinical performance thereby ensuring its suitability for diverse applications in research and clinical settings (Akinaga et al., 2024). This validation process focused on several critical parameters. Firstly, analytical performance was thoroughly evaluated to determine the assay’s precision, detection limit, linearity, and analytical specificity (Akinaga et al., 2024). Secondly, clinical performance was assessed to establish the assay’s sensitivity and specificity within a clinical context with mycobacterial culture used as the reference standard. Moreover, the correlation with bacterial load was investigated to understand the relationship between quantified LAM concentrations and the actual bacterial burden. Biosafety was of a paramount consideration with validation efforts specifically confirming the effectiveness of the heat inactivation step in rendering sputum samples non-infectious, thus, ensuring safe handling throughout the assay procedure.

### 3.2 Step-by-step procedures

#### 3.2.1 Specimen requirements

The PATHFAST TB LAM Ag assay has been validated in sputum as the primary specimen type. Emerging data support its application in other biological samples including urine, serum, plasma, stool, and exhaled breath condensate, though these applications remain preliminary and require further validation before routine clinical use (Pichlo et al., 2025; Fouchet et al., 2025; Kolokotroni et al., 2026). For the standardized protocol described here, sputum is the recommended specimen.

##### 3.2.1.1 Sputum and urine collection

Collect approximately 1–3 mL using standard morning early sputum collection or induced sputum collection. Place in a sterile, clear, plastic, leak-proof 50 mL screw-cap centrifuge tube. Transport immediately to the laboratory on ice and process within 24 hours.

Collect mid-stream urine in a sterile leak-proof container. Proof-of-concept for longitudinal urinary LAM monitoring using PATHFAST has been demonstrated across 24 weeks in pulmonary TB patients (Kolokotroni et al., 2026). Urine-based monitoring is preliminary and requires further validation, particularly in HIV-positive patients.

##### 3.2.1.2 Sample input options

The assay can be performed on either raw sputum or NALC-NaOH decontaminated sputum pellets. Both undergo the same NaOH-based LAM extraction procedure. The use of NALC-NaOH decontaminated pellets improves reproducibility (Pichlo, 2025; Pichlo et al., 2025). Decontaminated pellets also maintain LAM detectability after biobanking at −80 °C enabling retrospective analysis of stored samples (Orina et al., 2025).

##### 3.2.1.3 GTC incompatibility

GTC solution reduces detectable LAM by up to 98.3% and is likely incompatible with PATHFAST (Pichlo, 2025). If both PATHFAST and TB-MBLA are to be performed on the same patient, separate sputum aliquots with different processing protocols are required.

#### 3.2.2 Sample preparation

**Figure 1:**
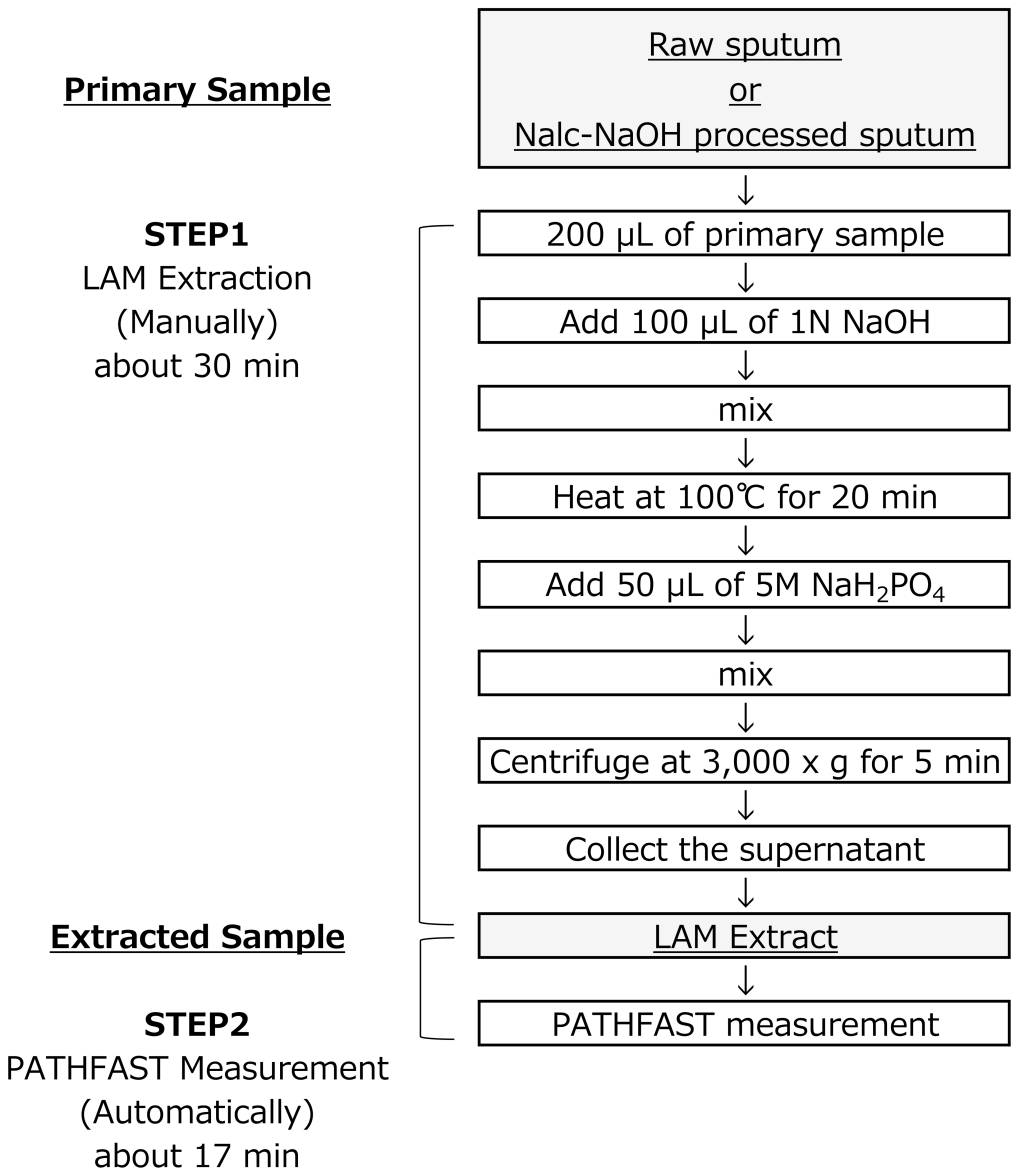
Standardized protocol for sputum sample processing and PATHFAST TB LAM Ag measurement. Schematic representation of the two-step workflow for the quantification of LAM using the PATHFAST system. Step 1 (Manual LAM extraction): 200 µL of raw or NALC-NaOH processed sputum is subjected to chemical lysis with 1N NaOH, followed by heat treatment at 100 °C for 20 minutes to release and solubilize the LAM antigen. The sample is subsequently neutralized with 5M NaH_2_PO_4_ and centrifuged at 3,000g for 5 minutes to isolate the supernatant containing the LAM extract. Step 2 (Automated measurement): The resulting extract is loaded into the PATHFAST analyzer for fully automated Chemiluminescent Enzyme Immunoassay (CLEIA) measurement. The total turnaround time from primary sample to result is approximately 47 minutes, comprising 30 minutes of manual preparation and 17 minutes of automated analysis.

Upon receipt, within a biosafety cabinet, 200 µL of the sputum sample, which is the validated volume for this protocol, is aliquoted into a 1.5 mL or 2.0 mL microcentrifuge tube (**Figure 1**). This is followed by heat inactivation, a critical biosafety step, where 100 µL of 1.0 N NaOH is added to the sputum sample, briefly vortexed for mixing, and then incubated in a heat block or water bath at 100 °C for 20 minutes (Akinaga et al., 2024). Ensure the heat block is pre-verified to reach 100 °C accurately before use. This heat inactivation step renders the sample non-infectious (BSL-1 post-extraction) as validated by Akinaga et al. (2024). The 20-minute duration must be strictly observed as incomplete heating poses a biosafety risk. After heating, the tube is removed and allowed to cool to room temperature before neutralization with 50 µL of 5 M NaH_2_PO_4_ followed by brief vortexing. The final step in sample preparation is centrifugation at 3,000 × g for 5 minutes, which separates the sample components leaving the extracted LAM in the supernatant (Akinaga et al., 2024).

#### 3.2.3 PATHFAST analysis

Prior to sample analysis, analyzer preparation is performed (Akinaga et al., 2024). The PATHFAST analyzer is powered on, and the system undergoes initialization and warm-up typically taking around 20 minutes. During this time, the MC ENTRY CARD is scanned for new reagent lots, and the waste box is prepared along with loading the required number of pipette tips. For sample loading, 100 µL of the supernatant, containing the extracted LAM, is carefully aspirated from the prepared sample tube and dispensed into the designated well of a PATHFAST TB LAM Ag reagent cartridge. Once loaded, the cartridge is placed into the PATHFAST analyzer to start the assay. The analyzer then automatically executes all subsequent steps, including incubation, washing, and signal detection, without further user intervention. After approximately 17 minutes, the result interpretation is complete, and the quantitative LAM concentration, reported in pg/mL, is displayed on the analyzer screen (Akinaga et al., 2024).

### 3.3 Precision, accuracy, and limits of detection

The PATHFAST TB LAM Ag assay demonstrates robust analytical performance across several key metrics (Akinaga et al., 2024). Regarding precision, the assay exhibits high reliability, with single-site repeatability coefficients of variation (CVs) ranging from 5.2% to 7.0%. Furthermore, multi-site reproducibility CVs were consistently low, between 7.1% and 8.4%, indicating excellent consistency across different laboratory settings. The accuracy of the assay is strongly supported by its clinical performance achieving a high sensitivity of 88.8% and a specificity of 100% when compared against the gold standard (Akinaga et al., 2024). Critical to its utility are the established limits of detection and quantification. The limit of blank (LoB) for the assay is 3.03 pg/mL, while the limit of detection (LoD) is precisely determined at 6.67 pg/mL (CLSI, 2012). The limit of quantification (LoQ) is 7.44 pg/mL established at a coefficient of variation of 20% (CLSI, 2012). Beyond these limits, the assay maintains excellent linearity up to a concentration of 50,000 pg/mL with recovery rates consistently falling within the acceptable range of 85% to 118%.

### 3.4 Expected outcome

The PATHFAST analyzer is designed to deliver a quantitative result for LAM concentration, expressed in pg/mL. This precise measurement can be effectively utilized to assess the bacterial load at any given time point and to meticulously monitor changes in bacterial load throughout the course of treatment. The analyzer has a significant throughput and is capable of processing up to 6 samples per run with a remarkably short measuring time of around 17 minutes. The system facilitates seamless data transfer through various standard communication protocols, including ASTM, Fixed standard, RS-232C enabling integration into laboratory information systems. A key advantage of this protocol is its rapid turnaround time. The entire process, from initial sample preparation to the final result, can be completed in less than one hour which is crucial for timely clinical decision-making. Moreover, the assay is anticipated to exhibit high sensitivity and specificity, with expected values exceeding 85% and 95% respectively when compared to mycobacterial culture.

### 3.5 Anticipated results

The expected outcome of this protocol is the successful and quantitative detection of LAM in sputum and other samples thereby providing a rapid and accurate measure of bacterial load in patients diagnosed with pulmonary TB. The results generated through this standardized procedure are anticipated to be highly reproducible and to demonstrate a strong correlation with conventional culture-based methods serving as a reliable indicator of disease activity and treatment response.

### 3.6 Example of application and effectiveness

This protocol has been successfully applied in a study evaluating the PATHFAST TB LAM Ag assay for monitoring treatment response in patients with pulmonary TB (Akinaga et al., 2025). The results showed a significant decline in LAM concentrations during the first 14 days of treatment, with a half-life of 8.3 days, which correlated well with the decline in bacterial load as measured by culture. This demonstrates the effectiveness of the protocol for quantitatively monitoring treatment response.

## 4 Discussion

The PATHFAST TB LAM Ag assay represents a significant advancement in the field of TB diagnostics, particularly treatment monitoring, addressing many of the limitations of current methods. The protocol presented here provides a standardized step-by-step procedure for the quantitative detection of LAM in biological samples enabling rapid and accurate detection and assessment of mycobacterial load. The key innovation of this assay is the combination of a rapid heat inactivation step, which enhances biosafety, with a fully automated measurement platform to reduce hands-on time and minimizes the potential for human error.

The PATHFAST TB LAM Ag assay occupies a distinct and currently unoccupied position in the LAM diagnostic landscape. Existing WHO-recommended urine LAM tests, including Determine TB-LAM (AlereLAM) and FujiLAM II (SILVAMP), provide binary or semi-quantitative results and are recommended specifically for HIV-positive patients with advanced immunodeficiency for diagnostic purposes (Bjerrum et al., 2019; Broger et al., 2019; Kerkhoff et al., 2020). The LAM-ELISA Otsuka provides quantitative sputum LAM measurement but requires over five hours and is not commercially available. PATHFAST is currently the only commercially available, CE-IVD certified, quantitative sputum LAM assay with a 47-minute turnaround, addressing the complementary and unoccupied use case of treatment monitoring in pulmonary TB patients regardless of HIV status.

The analytical performance of the PATHFAST TB LAM Ag assay is excellent, with high precision, a low detection limit, and a wide linear measurement range (Akinaga et al., 2024). These characteristics are crucial for a reliable quantitative assay as they ensure that the measurements are reproducible and can accurately reflect the true concentration of LAM in a sample. The clinical performance of the assay is also impressive with a sensitivity of 88.8% and a specificity of 100% when compared to culture (Akinaga et al., 2024). This level of accuracy meets the WHO’s minimal requirements for a new TB diagnostic test and approaches the optimal criteria (WHO, 2023; Gupta-Wright et al., 2023; Pichlo et al., 2025). The strong correlation between LAM concentration and bacterial load, as measured by time to detection in MGIT culture further validates the use of LAM as a biomarker for TB monitoring treatment response (Akinaga et al., 2024, 2025).

An independent evaluation found that the manufacturer cut-off of 10 pg/mL yielded 85.2% sensitivity but only 70% specificity in a European cohort (Pichlo et al., 2025). An optimized cut-off of 15.44 pg/mL restored specificity to 100% while maintaining 85.2% sensitivity (AUC = 0.90). These findings suggest that the cut-off may require population-specific optimization, and laboratories implementing this protocol should validate the cut-off in their local patient population before routine clinical use. A separate French evaluation reported 75% sensitivity and 95% specificity in 60 patients, a finding likely explained by the higher proportion of smear-negative patients in that cohort where LAM sensitivity is inherently lower (Fouchet et al., 2025).

One of the most significant advantages of the PATHFAST TB LAM Ag assay is its rapid turnaround time. The entire protocol, from sample preparation to result, can be completed in less than one hour compared to the two to eight weeks required for culture-based methods. This rapid turnaround time allows for timely clinical decision-making which can improve patient outcomes and reduce the risk of treatment failure and drug resistance. The heat inactivation step is another major advantage as it renders the sample non-infectious, reducing biosafety requirements and allowing for safer handling in a wider range of laboratory settings. This is particularly important in settings where access to BSL-3 facilities may be limited.

The PATHFAST analyzer is classified as suitable for primary care center level (Branigan, 2025). Operational requirements include stable electrical power, reagent refrigeration at 2–8 °C, 1–2 days of operator training, and periodic service calibration. The current commercial price is US$32–54 per test (Branigan, 2025). This is above the WHO monitoring TPP indicative threshold (WHO, 2023; Gupta-Wright et al., 2023).

The protocol has been successfully applied in several clinical studies demonstrating its effectiveness for monitoring treatment response in patients with pulmonary TB using sputum and urine samples (Akinaga et al., 2024, 2025; Fouchet et al., 2025; Orina et al., 2025; Kolokotroni et al., 2026). In these studies, the PATHFAST TB LAM Ag assay was able to track the decline in LAM concentrations during treatment which correlated well with the decline in bacterial load as measured by culture. This demonstrates the utility of the assay for quantitatively monitoring treatment response and for evaluating the early bactericidal activity of new TB drugs.

However, there are some limitations to the PATHFAST TB LAM Ag assay that should be considered. In the analytical validation study by Akinaga et al. (2024), 178 NTM strains were evaluated, and 79.1% of the slow-growing NTMs (e.g., *M. avium* complex, *M. intracellulare*, *M. kansasii*, and *M. xenopi*) showed cross-reactivity, while 85.9% of rapid-growing NTMs (e.g., *M. abscessus* and *M. smegmatis*) showed no cross-reactivity. In settings where NTM pulmonary disease is prevalent, a positive PATHFAST result must be interpreted in clinical context. Furthermore, confirmatory NTM species identification is recommended when NTM infection is suspected. This cross-reactivity profile may also forms the biological basis for the emerging application of PATHFAST for NTM pulmonary disease treatment monitoring (Fouchet et al., 2025). The quality of the sample can also affect the accuracy of the results. Sputum samples with low viscosity or high levels of contaminants may be difficult to process. To address this issue, it is recommended that samples be homogenized with a 2.5% NaOH-NALC solution prior to processing, which has been shown to improve the reproducibility of the assay (Pichlo et al., 2025).

The reproducibility of PATHFAST measurements is substantially influenced by pre-analytical sample processing (Pichlo, 2025; Pichlo et al., 2025). Non-homogenized raw sputum yielded a mean coefficient of variation (CV) of 54.51% ± 31.75% in duplicate measurements which is clinically unacceptable for quantitative serial monitoring (Pichlo, 2025; Pichlo et al., 2025). Decontamination of sputum using NALC-NaOH solution prior to the NaOH-based LAM extraction reduces the mean CV to 16.0% ± 25.0%, with samples above the limit of quantification consistently achieving CV <20% (Pichlo, 2025; Pichlo et al., 2025). These findings support the use of NALC-NaOH decontaminated sputum pellets as the preferred input for serial treatment monitoring applications.

GTC solution, used in TB-MBLA sample processing, was found to substantially reduce detectable LAM in sputum (Pichlo, 2025). Two sputum samples processed using GTC working solution showed pronounced LAM reductions from 19,667 pg/mL to 2,237 pg/mL (88.6% reduction) in the first sample and to just 1.7% of the untreated concentration (98.3% reduction) in the second sample (Pichlo, 2025). Although based on only two samples, these findings indicate that GTC-based processing is incompatible with PATHFAST LAM detection. Laboratories running both PATHFAST and TB-MBLA on the same patient must collect separate sputum aliquots and process them with their respective protocols.

The clinical performance of the PATHFAST TB LAM Ag test was evaluated in a FIND biobank cohort (n=100: 80 culture-positive, 20 culture-negative sputum specimens from untreated pulmonary TB patients) (Akinaga et al., 2024). The evaluation demonstrated 88.8% sensitivity (95% CI: 80.0–94.0%) and 100% specificity (95% CI: 83.9–100%) compared to mycobacterial culture as the reference standard (Akinaga et al., 2024). A strong quantitative correlation was observed between PATHFAST-measured LAM concentration and bacterial load as measured by MGIT time-to-detection (Spearman r = −0.770), independently confirmed in the German DZIF TB cohort (Spearman rho = −0.54; 95% CI: −0.79 to −0.15; p = 0.009) (Pichlo et al., 2025).

To ensure optimal performance and reliable results, several potential pitfalls and troubleshooting measures should be considered. Incomplete heat inactivation represents a significant biosafety risk. If the sample is not heated to 100 °C for the full 20 minutes, inactivation may be incomplete. It is imperative to ensure that the heat block or water bath is accurately calibrated and that the entire heating duration is strictly observed. Similarly, incomplete neutralization of the sample after NaOH treatment can adversely affect assay performance. Proper neutralization requires adding the correct volume of neutralization buffer and ensuring thorough mixing of the sample. Lastly, the PATHFAST analyzer may occasionally display analyzer errors. In such instances, users should consult the instrument’s user manual for specific troubleshooting guidance and resolution steps.

The validated input volume for this protocol is 200 µL of sputum (Akinaga et al., 2024). No data currently exist on the use of smaller input volumes for paediatric patients or specimens of limited volumes. This represents a gap that requires dedicated validation studies, particularly given the importance of TB monitoring in paediatric populations. Moreover, the reported multi-site reproducibility CV of 7.1–8.4% was obtained under controlled study conditions. In routine clinical laboratory settings, operator-dependent steps, particularly viscous sputum aspiration, may introduce additional variability not fully captured in the reported CVs. The use of a dedicated high-density liquids pipette (Microman M1000E or equivalent) is mandatory and is the principal mitigation for operator variability. NALC-NaOH decontaminated sputum pellets further reduce sample heterogeneity and are strongly recommended for serial monitoring applications requiring reproducible absolute values across time points.

In conclusion, the PATHFAST TB LAM Ag assay is a promising new tool for TB treatment monitoring that offers several advantages over existing methods, including rapid turnaround time, high sensitivity and specificity, and enhanced biosafety. The comprehensive protocol presented in this paper provides a solid foundation for the implementation of this assay in both clinical and research settings. Further research is needed to validate the assay in different populations, such as people living with HIV and children, and to explore its utility in non-sputum sample types. However, the evidence to date suggests that the PATHFAST TB LAM Ag assay has the potential to make a significant contribution to the global fight against tuberculosis.

## Data Availability

All data discussed in this study can be found within the article and are available in the previously published studies cited herein (Akinaga et al., 2024, 2025; Pichlo, 2025; Pichlo et al., 2025; Orina et al., 2025; Fouchet et al., 2025; Kolokotroni et al., 2026). Further inquiries can be directed to the corresponding author.
